# Modeling and Analyzing Gene Co-Expression in Hepatocellular Carcinoma Using Actor-Semiotic Networks and Centrality Signatures

**DOI:** 10.4137/cin.s1043

**Published:** 2008-11-05

**Authors:** David C.Y. Fung

**Affiliations:** School of Information Technologies, The University of Sydney, Sydney, New South Wales 2006, Australia

**Keywords:** actor-semiotic network, node centrality, graph signature, gene co-expression, hepatocellular carcinoma

## Abstract

Primary hepatocellular carcinoma (HCC) is currently the fifth most common malignancy and the third most common cause of cancer mortality worldwide. Because of its high prevalence in developing nations, there have been numerous efforts made in the molecular characterization of primary HCC. However, a better understanding into the pathology of HCC required software-assisted network modeling and analysis. In this paper, the author presented his first attempt in exploring the biological implication of gene co-expression in HCC using actor-semiotic network modeling and analysis. The network was first constructed by integrating inter-actor relationships, e.g. gene co-expression, microRNA-to-gene, and protein interactions, with semiotic relationships, e.g. gene-to-Gene Ontology Process. Topological features that are highly discriminative of the HCC phenotype were identified by visual inspection. Finally, the author devised a graph signature-based analysis method to supplement the network exploration.

## 1. Introduction

Primary hepatocellular carcinoma (HCC) is the fifth most common malignancy and the third most common cause of cancer mortality worldwide with one million new cases diagnosed annually. Its prevalence is much higher in developing nations than in industrialized nations. At present, 80% of the HCC cases came from the East Asia and the sub-Saharan Africa with China accounting for nearly 55% of them [1]. For this reason, there have been numerous efforts made in the molecular characterization of primary HCC. As a result, there is a rich repository of genomic and proteomic data available for public access [2]. To uncover the biology hidden within such a large volume of data will require software-assisted network modeling and analysis (reviewed in [3]). In recent years, attempts to characterize disease phenotypes by integrative network modeling and analysis have been made. For example, Tuck et al. [4] retrieved the human gene regulatory network from the TRANSFAC^®^ database and integrated it with the transcription factor-to-target genes co-expression network derived from multiple microarrays. They then demonstrated that node degree measures are a feasible discriminator of oncology types. Chuang et al. 5 characterized proteomic sub-networks as the biomarkers for discriminating between metastatic and non-metastatic breast cancer. They demonstrated that the protein sub-networks identified are highly discriminative of metastasis and some of the genes underscored by statistical inference methods were found to be member nodes of those sub-networks. These studies demonstrated the effectiveness of network modeling and analysis.

This paper presents the author’s first attempt in exploring the biological implication of gene co-expression in HCC using actor-semiotic network modeling. The rationale was that a complex network requires context or metadata to be comprehensible. Without which, no human user would be able to unpack the information content within, let alone making biological deductions. The proposed *actor-semiotic* network is similar to the *actor-network* 6 frequently used for modeling healthcare systems. Actor-network theory models the human community as a network of heterogeneous actor-semiotic interactions. The actors are human participants, human organizations, and material objects. The semiotics is the human ideas, concept, and policies. In molecular biology, the actors are the bio-molecules and the sub-cellular components. The semiotics is the human understanding of biology. Its abstraction is the ontologies on biological processes, molecular function, and cellular phenotypes.

Because the topology of an actor-semiotic network is determined by the combination of inter-actor and semiotic relationships, there should be visually identifiable topological features that are highly discriminative of the HCC phenotype. To achieve this, the author employed visual inspection and, in addition, a graph signature-based analysis method to supplement network exploration. This method first summarized the local topology of every node in the network as a signature vector and then projected the vectors onto a two-dimensional scatterplot for further exploration.

## 2. Topological Analysis of the Actor-Semiotic Network

### 2.1. Visual analysis

Using NetMap Decision Director™, an actor-semiotic network *G* (|*V*| = 9313; |*E*| = 49,393) was being constructed. *G* was a union of all the actor and semiotic nodes and edges described in section 6.2. The bio-molecules and the sub-cellular components within *G* were represented by the actor nodes whereas the biological context of *G* was represented by the semiotic nodes (see Appendix A.1). The pairwise interactions between bio-molecules or between bio-molecules and sub-cellular components were represented by the inter-actor edges. The ontological relationships between actor and semiotic nodes were represented by the semiotic edges.

**Figure 1:**
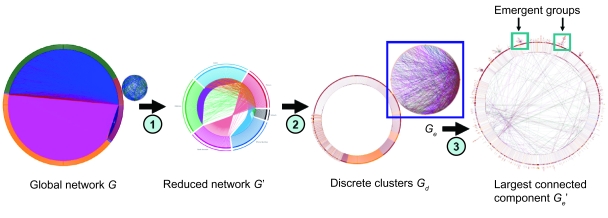
Exploring the actor-semiotic network of HCC. (1) The network *G* was reduced to a smaller network *G*′ by excluding extraneous *Protein* nodes and *Gene Ontology* nodes that did not map to the *Gene* nodes in the co-expression network. (2) *G*′ was transformed to *G_d_* to expose any nested discrete clusters. (3) The largest connected component *G_e_*′ was extracted from *G_e_* which is the largest cluster in *G_d_*. Node centrality signature vectors of *G_e_*′ were constructed before biological inference.

**Figure 2:**
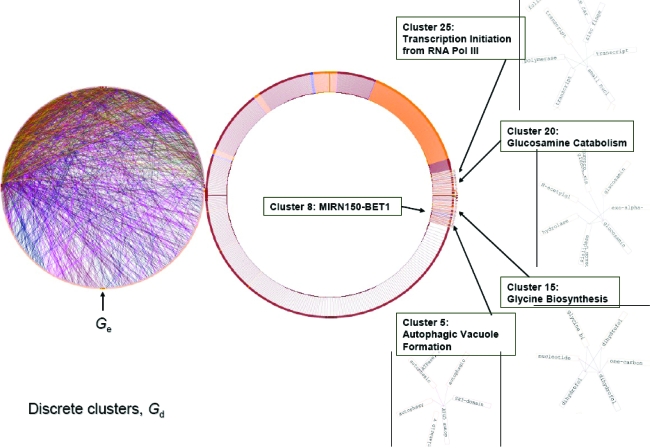
Network topology of *G_d_*. Gene co-expression in the normal hepatocyte is represented by green-coloured edges whereas co-expression in the hepatocellular carcinoma is represented by dark red-coloured edges. Gene nodes are coloured dark red. *GO* nodes are coloured yellow. *MIRNA* nodes are coloured blue.

A smaller network *G*′ (|*V*| = 1668; |*E*| = 2473) was derived from *G* as a result of node mapping (see Appendix A.2 and Fig. 1). To test whether *G*′ contained a set of nested networks, it was decomposed to *G_d_* using NetMap™. The nested networks observed in *G_d_* are discrete clusters. Let *C_k_* be one of the clusters, then *G_d_* = {*C_k_*} where 0 < *k* ≤ 32 (Fig. 2). Each cluster is a network that is not connected to any other clusters nor does it share any of its member nodes with other clusters, such that *C_i_* ∩ *C_j_* = 0 for *C_i_*, *C_j_* ⊆ {*C_k_*} where *i* ≠ *j*. Although their size |*V*| ranged from 2 to 1536, only one cluster had a |*V*| of 1536. The rest had a |*V*| that ranged from 2 to 7. Among the inter-actor edges in the small clusters (1 < |*V*| < 8), only 10 were of the Coexpression_ HCC subtype and 24 were of the Coexpression_liver subtype. It showed that most co-expressed genes, whether in the normal hepatocyte or in HCC, are highly inter-connected. Eight of the small clusters contained only semiotic edges. For the small clusters that contained at least one inter-actor edge, the semiotic nodes indicated that the protein-coding genes within each cluster shared the same biological process or molecular function (Fig. 2).

**Figure 3:**
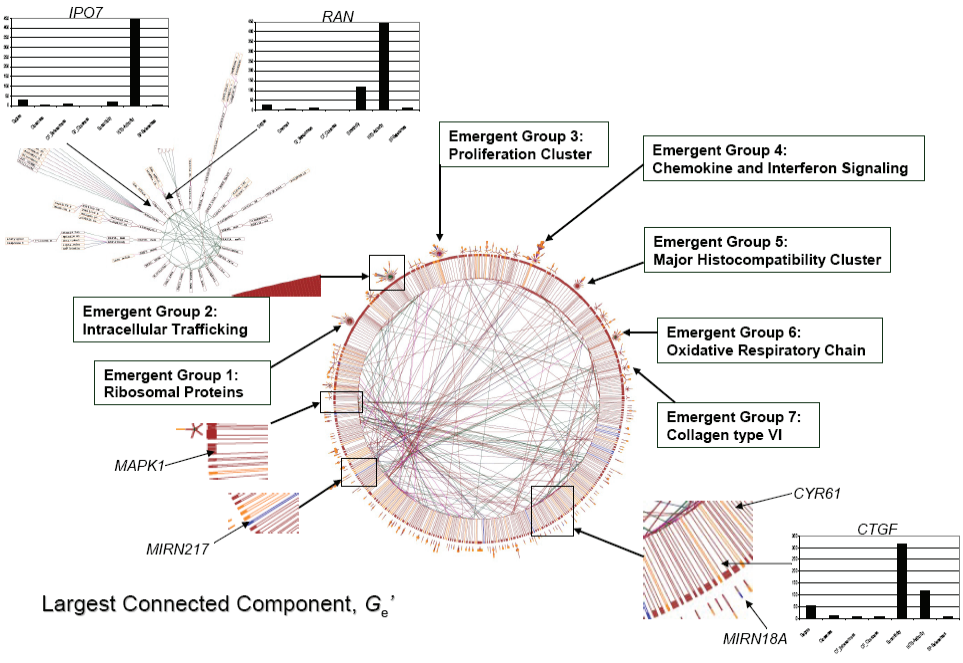
Network topology of *G_e_*′. The colour coding used for nodes and edges is the same as in Figure 2. The rank scores for the seven centrality types in each bar chart are arranged (from left to right) in this order: Degree, Closeness, Current Flow-Betweenness, Current Flow-Closeness, Eccentricity, HITS-Authority, and Shortest Path-Betweenness. A lower rank score means a higher node ranking for a particular centrality type.

From the largest cluster *G_e_* (|*V*| = 1536; |*E*| = 2367) in *G_d_*, the largest connected component *G_e_*′ (|*V*| = 1371; |*E*| = 1120) was extracted (Fig. 1). *G_e_*′ was comprised of inter-connected emergent groups and liaison nodes (Fig. 3). An emergent group is a sub-network in which its member nodes are more inter-connected within than without. A liaison node is a node shared by multiple emergent groups. The emergent groups were localized in the top half and the liaison nodes in the lower half of *G_e_*′. Of the 41 emergent groups, six of them had a |*V*| larger than 25. The semiotic edges within each emergent group indicated that it belongs to a specific biological process showing that the topology of the integrated co-expression and protein interaction network in HCC is partially modular. In a sense, each emergent group is similar to the Complex Biological Module proposed by Zotenko et al. 30. Some emergent groups, e.g. groups 4 and 5, are directly linked to one another suggesting that the coupling between their corresponding biological processes could be hard-wired. Some, e.g. groups 2 and 3, are connected via liaison nodes, *MAPK1* and *MIRN217*, suggesting that the coupling between their corresponding biological processes could be switch-dependent.

### 2.2. Network exploration using graph signatures

**Figure 4:**
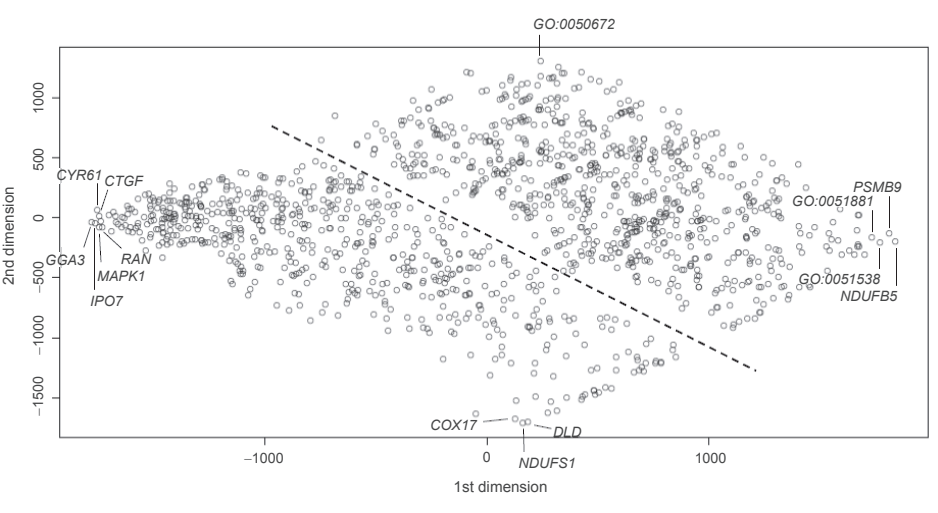
Scatterplot generated by projecting graph signatures of *G_e_*′ to the 2D space using Kruskal’s multi-dimensional scaling.

The eccentricity and radiality centralities were found to give identical rankings. The same was also observed with the HITS-Authority and HITS-Hub centralities. Therefore the radiality and the HITS-Hub centralities were excluded from the signature vector of each node. After the signature vectors were computed and scaled, the scatterplot shows that there are two clusters of nodes, each representing a different range of signature vectors (Fig. 4). Nodes within the emergent groups were found in the upper cluster and liaison nodes were found in the lower cluster.

The six nodes at the left-extremity (x-range = [−1661.93, −1617.66]; y-range = [−74.14, 61.57]; Fig. 4) of the lower cluster have signatures that contained the top 5% ranking in closeness, current-flow betweenness, current-flow closeness, and shortest-path betweenness centralities. Three of these nodes *GGA3*, *IPO7* and *RAN* are members of emergent group 2 (Fig. 3). They are involved in intracellular trafficking. Another two, *CTGF* and *CYR61* are liaison nodes involved in angiogenesis. The last one, *MAPK1* is also a liaison node which is an amplifier shared by multiple signal transduction pathways. The four nodes at the right-extremity (x-range = [1620.29, 1719.54]; y-range = [−125.71, −184.45]; Fig. 4) of the upper cluster have signatures that contain the bottom 10% ranking in all seven centrality types. Two of them, *NDUFB5* and *PSMB9*, are actor nodes. Another two, GO:0051538 (3 iron, 4 sulfur cluster binding) and GO:0051881 (regulation of mitochondrial membrane potential), are semiotic nodes. *NDUFB5* is a subunit of the ubiquinone complex in the mitochondrial electron transport chain whereas *PSMB9* is a proteosome subunit. Both proteins are peripheral nodes in the emergent group 6 (Fig. 3).

The three nodes at the bottom corner of the lower cluster (x-range = [119.32, 173.93]; y-range = [−1570.32, −1597.41]; Fig. 4) have signatures that contain the bottom 10% ranking in closeness, current-flow closeness, eccentricity, and HITS-authority centralities, and the top 10% ranking in degree, current-flow betweenness, and shortest-path betweenness centralities. They are *COX17*, *NDUFS1*, and *DLD*. Each of these nodes was a junction to two small subsets of nodes with each subset containing a maximum of three nodes. Yet at least one node within each subset was connected to another two nodes without. *COX17* and *NDUFS1* are subunits of the mitochondrial electron transport chain with the latter being another subunit of the ubiquinone complex. *DLD* is a subunit of the pyruvate dehydrogenase complex. The semiotic node at the top corner of the upper cluster (x = [225.65]; y = [1223.89]; Fig. 4) is GO:0050672 (negative regulation of lymphocyte proliferation). It had a signature that contain the bottom 10% ranking in degree, current-flow betweenness, and shortest-path betweenness centralities, the top 10% ranking in closeness and eccentricity centrality, and the top 5% ranking in HITS-authority centrality.

In summary, the ranking of all centralities decreases as one moves to the right end of the x-axis in the scatterplot. On the other hand, the node ranking on degree, current-flow betweenness, and shortest-path betweenness centralities increase as one moves to the lower end of the y-axis but at the same time, the rankings on closeness, currentflow closeness, eccentricity, and HITS-authority centralities decrease. The rank score of those nodes mentioned in this paper are tabulated in Table 1.

## 3. Inference of HCC Biology

Based on the visual exploration of network *G_e_*′ and the inspection of the scatterplot, the author deduced several hypotheses on the molecular pathology of HCC as described in the following sections. Since cell cycle events have been well studied in recent years, emergent group 3 was used to demonstrate that the actor-semiotic network is a model consistent with the current knowledge on cell proliferation. MicroRNAs have recently been discovered as new players in regulating oncogenic signal transduction. In section 3.2, the author hypothesized the influence of *MIRN18A* on angiogenesis in HCC and how this could contribute to tumor invasiveness. Also gaining attention lately is the role of intracellular trafficking in establishing the malignant phenotype. In section 3.3, the author hypothesized the possible effect of nuclear export disruption on growth factor-induced gene regulation.

### 3.1. De-synchronized cell cycle phases

The semiotic nodes in emergent group 3 indicated that it contains exclusively cell cycle genes (Fig. 3). Their co-expression was found only in HCC and could be a result of replication stress. Within this emergent group, *UBE2C* has the highest node degree centrality. Of interest, *UBE2C* up-regulation has frequently been observed in a variety of malignancies including HCC [2, 7]. *UBE2C* was found to link with three semiotic nodes, GO:0007051 (spindle organization), GO:0008054 (cyclin catabolism), and GO:0031536 (positive regulation of mitotic exit), as compared to only one or two seen among its co-expressed neighbours. Hence *UBE2C* is functionally more diverse but still operates exclusively within the cell cycle. This agrees with the consensus that *UBE2C*, an E2-ubiquitin conjugating enzyme, is a subunit of the anaphase promoting complex (APC/C) which mediates substrate ordering 8. Substrate ordering refers to the proper sequence of protein ubiquitination that ensures the orderly degradation of different proteins during cell cycle progression. It has been known that APC/C inactivation is mediated by *UBE2C* auto-ubiquitination, a result of *UBE2C* up-regulation 9. If this up-regulation is persistent in HCC, APC/C inactivation could be prolonged beyond the S phase. One probable effect would be the reduction in cyclin catabolism which could lead to a shortened G1 phase and a prolonged S phase due to the disruption in DNA replication 10. With the loss of substrate ordering, the author hypothesized that the cell cycle phasing would be de-synchronized. Molecular events that are S phase specific could co-exist with those in the G2 and M phases. Eventually, mitotic exit could be delayed or even failed.

### 3.2. Abnormal angiogenesis

*CYR61* (*CCN1*) and *CTGF* (*CCN2*) were found to co-express with *TGFB1* in HCC only. Both belong to the *CCN* family of immediate early genes activated by TGFβ1 11 and by hypoxia 12. Previous work suggested that *CYR61* induces endothelial cell proliferation, cell adhesion, and angiogenesis through the activation of integrin (*ITGAV*-*ITGB3* complex) expression 13. *CTGF* induces the secretion of collagen and fibronectin which form the scaffolding of the extracellular matrix, a step crucial to the formation of a neo-vasculature 14. That explained why it is directly linked to *COL6A1* and *COL6A3* in emergent group 7. As shown in the lower right inset of Figure 3, *CTGF* is a predicted target of *MIRN18A*. This microRNA gene, which has been found to express in some Japanese HCC patients, is both liver- and tumor-specific 15. The author hypothesized that the expression of *MIRN18A* in HCC could lead to matrix instability due to the reduced translation of *CTGF* transcripts. The dynamics of angiogenesis could therefore be altered if the molecular abundance of *CYR61* is higher than that of *CTGF*. One consequence could be excessive endothelial cell migration and proliferation but inadequate cell anchorage due to an unstable extracellular matrix and hence poor tubular formation. Tumor vasculature is known to be structurally chaotic with excessive leakage 16 and *MIRN18A* expression could be a contributing factor. This may enhance HCC metastasis in two ways. The first could be enhanced tissue invasion by *MMP*s induced by the hepatitis B viral oncoprotein *HBX* in malignant cells 17. The second could be the intravasation of malignant cells into the neo-vasculature but also rapid extravasation to the surrounding tissue because of vascular leakage.

### 3.3. Disrupted nuclear transport

*IPO7* and *RAN* were found to co-express not only with each other but also with nine other protein-coding genes (emergent group 2; Fig. 3). The semiotic nodes indicated that they are all involved in intracellular trafficking. Their co-expression occurred only in normal hepatocytes suggesting that intracellular trafficking could be aberrant in HCC. One possible cause could be the disruption of nucleocytoplasmic trafficking by *HBX*. Specifically, *HBX* disrupts nuclear export by sequestrating the export receptor *XPO*. Furthermore, the nuclear import and export processes require the GTPase protein *RAN*. It controls the interaction of *XPO* and of the importin receptor *IPO7* with their target proteins 18. If the majority of the *XPO* in HCC is being inactivated by *HBX*, it is possible that there will be a surplus of *RAN* available for mediating nuclear import by *IPO7*.

Recent findings revealed that many growth factors, e.g. *CTGF*, *CYR61*, *EGF*, *FGF*, *IFNG*, and their cell surface receptors can be endocytosed, then imported into the nucleus by importin receptors, and eventually exported by exportin receptors (reviewed in 19). Within the nucleus, they interact with various transcription factors, e.g. *E2F1* and *STAT3*, or co-regulators 20. Apart from regulating the transcription of specific target genes, they could also be involved in DNA replication 21 and repair 22, and RNA metabolism 23. Therefore the author hypothesized that the *HBX*-induced imbalance between nuclear import and export volumes could prolong growth factor activities inside the nucleus. Already, there have been studies suggesting that, at least for *FGF*s and *EGF*s, prolonged nuclear localization is correlated with cancer progression, resistance to radiotherapy and consequently poor prognosis 24.

## 4. Discussion

### 4.1. Strength and limitations of network analysis

Network analytics is very suited to biomedical research where high informational granularity and connectivity between objects are required for knowledge inference. However, the scale of the network often presents a cognitive challenge to the analyst. This limitation is partly moderated with the use of NetMap™ which allows the analyst to downsize a large network (|*V*| > 5000; |*E*| > 5000) by excluding nodes and edges selectively and then extract any sub-networks for further analysis. The 2D-projection of graph signatures further moderates the challenge of scale by providing a visual summary on the surrounding topology of every node in the form of a scatterplot. Using the latter as a guide, the analyst can then prioritize the nodes that need to be inspected first. At present, the author is testing this approach with networks that contained human disease terms 25 and cellular quiescence phenotypes 26 as semiotic nodes to see if one can discover more insights into the molecular pathology of HCC.

### 4.2. Biological implication of node centrality

There have been several views on how node centralities signify the biological essentiality of a protein. The first view took degree centrality as the primary indicator of biological essentiality because high degree protein nodes, also known as hubs, are essential for maintaining network connectivity 27. The second view argued that shortest-path betweenness centrality is a better indicator of essentiality 28. This view suggested that bottleneck proteins linked to multiple protein hubs are also biologically essential. The positive correlation between node degree and biological essentiality has been confirmed recently [29, 30] but the original rationale has been challenged 30. Zotenko et al.’s 30 proposition was that the hubs are essential because they form modules in which the member proteins are highly inter-connected and share a common biological function. They named the module as Essential Complex Biological Module (ECOBIM) because it is enriched in essential proteins. Furthermore, the authors demonstrated that current flow betweenness and shortest-path betweenness centralities are better indicators of connectivity, thus supporting the second view. So far, the above hypotheses were deduced from the yeast protein interaction network [27, 28, 30] and the human disease gene network 29 but how do they contribute to the current understanding of cancer biology?

The first view seemed to agree with the recent suggestion that it could take three mutated genes or fewer to induce early stage malignancy 31 since some well studied cancer genes, e.g. *APC*, *TP53*, *PTEN*, and *CDKN2A*, have a degree centrality greater than 20 (see Fig. 2 in 32). Further supporting evidence is that these genes are known to associate with familial cancers 33. However, Goh et al. 29 demonstrated that the vast majority of disease genes do not encode proteins high in degree centrality and therefore are not essential except for diseases that are fatal *in utero*. If applied to oncology, that will suggest that carcinogenesis does not necessarily involve genes (or proteins) of high degree centralities.

In the network *G_e_*′, the author observed that protein-coding genes that rank within the top 2% in degree centrality are not necessarily highly ranked in betweenness centralities. The best example comes from emergent group 1 in which *RPL6*, *RPL9*, *RPL14*, *RPL15* and *RPL31* rank within the top 2% in degree centrality but rank below 149th in current-flow centrality and rank below 100th in shortest-path betweenness centrality (Table 1). These genes are essential because RNA biosynthesis is fundamental to viability. The deletion of any one gene will affect the connectivity within the emergent group 1 more than without. This observation is in agreement with Zotenko et al.’s view. On the other hand, genes that rank within the top 10% in degree centrality and also within the top 5% in closeness, current-flow closeness, current-flow betweenness, and shortest-path betweenness centralities, are involved in signal transduction or intracellular trafficking suggesting that they could be the key drivers of disease progression if not carcinogenesis. Some of these proteins, e.g. *CXCR4*, *RAN* and *IPO*, are not only nodes within individual emergent groups but are also connected to liaison nodes and nodes of other emergent groups. Furthermore, a few signal transduction proteins, e.g. *CXCR4* and *MAPK1*, have degree centralities that rank within the top 2% and their current-flow betweenness and shortest-path betweenness centralities ranking within the top 1%. They are likely to be signaling hubs 32. Therefore, genes involved in HCC can have a high degree centrality but they can also serve as bottleneck proteins to multiple emergent groups. This deduction further refines Goh et al.’s proposition.

Thus far, none of the microRNA nodes found in *G_e_*′ are emergent group nodes but are liaison nodes. Their degree centralities rank between 252nd to 1314th with a median ranking of 569th. If projecting from Goh et al.’s and Zotenko et al.’s proposition, microRNAs are non-essential implying that their deletion may not be lethal but can contribute to abnormalities. Of the 15 microRNAs in *G_e_*′, four of them rank within the top 3% in closeness centrality. They are *MIRN148A*, *MIRN148B*, *MIRN217*, and *MIRN375*. The first two also rank within the top 2% in eccentricity. In addition, *MIRN217* rank within the top 2% in shortest-path betweenness centrality and *MIRN375* rank within the top 3% in current-flow betweenness and shortest-path betweenness centralities (Table 1). They share the common topological feature of being connected to liaison nodes on one side and emergent group nodes on the other side. Their ranking in the betweenness centralities seems to depend on the number of interaction partners and the node degree of each interaction partner. Based on the visualized topology and centrality rankings, it is reasonable to hypothesize that microRNAs which target signal transduction proteins or transcription factors of high degree, closeness, and betweenness centralities will exert the highest impact on the regulation of gene expression. This deduction seemed to agree with Cui et al.’s 34 proposition that the expression of the output layer genes in the signaling network is heavily regulated by microRNAs. Because the signal transduction network is inter-connected with the gene regulatory network 35, some proteins at the output layer could be bottlenecks that bridge the two networks and therefore are most likely to have high degree centralities as well as betweenness centralities.

## 5. Conclusion

The use of actor-semiotic network modeling and analysis does provide insight into the pathology of HCC. Although the inclusion of semiotic nodes increases the size of a network, they are useful for identifying discrete clusters or emergent groups that serve a particular biological process or a set of inter-related molecular functions. The provisions of network decomposition and sub-network extraction functionalities by NetMap™ facilitated the ‘top down’ exploration of a large graph. The use of graph signatures further facilitated network exploration by providing a summary of node topologies in a form of a scatterplot.

## 6. Methods

### 6.1. Data sources

#### Gene expression data

The gene co-expression profiles of HCC and normal hepatocytes were obtained from Gamberoni et al. 36 which was derived from the original dataset published by Chen et al. 37. A set of co-expressed genes from each sample set (normal hepatocyte or HCC) was extracted based on their Pearson’s correlation coefficients (*PCC* ≥ 0.86). This level of correlation, according to the random matrix theory, should be adequate for differentiating between the true co-expression modules and random noise 38.

#### MicroRNA expression data

The microRNA expression data of HCC and adjacent normal hepatocytes was published by Murakami et al. 15. The predicted microRNA target genes were curated from three publications [39–41].

#### Gene Ontology

The three categories of GO— Component, Process, and Function, were obtained from the Gene Ontology Consortium 42.

#### Human proteome data

The canonical human proteomic interaction data was obtained from the BioGrid version 2.0.36 43. This was integrated with the Hepatitis B-to-human proteomic interaction data obtained from the NCBI Gene RIF.

### 6.2. Data-to-network mapping

A relational database was constructed for storing the above datasets. Data for the edges were stored in four tables with each storing data of a specific edge type. The mapping of data to nodes and edges was done with the use of NetMap Decision Director™. The actor nodes are *GO Component*, *Gene*, *MIRNA*, and *Protein*. The semiotic nodes are *GO Process* and *GO Function*. The semiotic edges are of the type *Gene_To_GO* (Process or Function). Inter-actor edge types are *Gene_To_GO* (Component), *Gene_To_Gene*, *miRNA*_*To*_*Gene*, and *Gene_To_Protein. Gene_To_Gene* has two subtypes: Coexpression_HCC and Coexpression_Liver. *Gene_To_Protein* also has two subtypes: Human_Protein_Interaction and HBV_Human_Interaction.

### 6.3. Network visualization and interactivity

The visualization for the networks described in this paper was generated with the use of NetMap™. The software also allows the analyst to (1) decompose a large graph into a set of discrete clusters; (2) extract the largest cluster and identify its largest connected component; (3) decompose the largest connected component to inter-connecting emergent groups; (4) navigate from point-to-point within each network; and (5) search nodes by Gene Symbols or GO identifiers.

### 6.4. Emergent groups

The identification of emergent groups was completed by a proprietary pattern recognition algorithm embedded in NetMap™. These groups are so named because they *emerge* out of a given set of pairwise relationships. Hence, in a biological or social network, emergent groups are network structures that emerge out of local interactions 44. The NetMap™ algorithm was employed to examine the topology and the edge types of the relevant network and emergent group nodes were identified based on three criteria:

Given an emergent group *C_e_*(*V_e_*, *E_e_*),

- |*V_e_*| > 2
- *E_e_* = *V_e_* × *V_e_* such that |*E_e_*| > 2.
- Each node *v* ∈ *V_e_* has at least 50% of its edges connected to other nodes within *C_e_*.

Under these criteria, *C_e_* often appears as a subnetwork of high curvature which is the local density of triangular relations. Given that the curvature of a node, *curv*(*v*), is defined as:

curv(v)=t/n(n-1)/2

where *curv*(*v*) = [0, 1], *t* is the number of triangles, and *n* is the number of neighbours to node *v* 45, *curv*(*v*)→1 in *C_e_*.

### 6.5. Centrality measures

Node centralities are metrics for measuring the connectivity pattern of a node in relation to its surrounding neighbours. In this study, nine types of node centralities were calculated using CentiBiN 46. They are closeness, current-flow betweenness, current flow closeness, degree, eccentricity, HITS-authority, HITS-hub, radiality, and shortest-path betweenness centralities. The rationale behind each measure can be found in 47.

### 6.6. Signature vectors

After computing each node centrality type, the nodes were ranked in the descending order of their centrality values. The node with the highest value for, say degree centrality, would be assigned a rank score of 1. Hence the lower is the rank score, the higher is the node ranking for a certain centrality type. This step generated a column vector *R* = [*c_i_*] for each centrality type in which each entry c*_i_* is the rank score for node *i*. The iteration of the previous step generated a set of column vectors *S* = (*R*_0_, *R*_1_, …, *R_j_*) which formed the matrix *M* = [*c_ij_*] in which each entry *c_ij_* is the rank score for node *i* of the centrality type *j*. The node *i* can be an actor or a semiotic node. The signature vector *V_i_* for node *i* is defined as *V_i_* = (*c_i_*_0_, *c_i_*_1_, …, *c_ij_*) which is the row*_i_* of *M*. The matrix *M* was further factorized to give a smaller matrix *M*′ = [*c_ik_*] for *k* < *j* if some of the column vectors in *M* were identical. The resulting signature vector *V*′*_i_* for node *i* is therefore the row*_i_* of *M*′. Using Kruskal’s multi-dimensional scaling, the set of signature vectors {*V*′*_i_*} was then projected to a 2D space and visualized as a scatterplot 48.

### 6.7. Software availability

The NetMap Analytics™ software suite which includes NetMap Decision Director™ and NetMap™ is available from NetMap Analytics Proprietary Limited, Sydney, Australia (http://www.netmapanalytics.com.au) under an academic license.

## Figures and Tables

**Table 1:** Node centrality ranking of actor and semiotic nodes in *G_e_*′.

Node name	Degree	Closeness	Current flow betweenness	Current flow closeness	Eccentricity	HITS authority	Shortest path betweenness
*CTGF*	56	11	7	10	317	119	9
*COL6A1*	166	139	359	102	463	228	467
*COX17*	167	1285	62	1292	1270	1313	33
*CDKN2A*	165	271	21	281	159	221	39
*CYR61*	95	7	29	20	162	150	6
*CXCR4*	3	34	5	16	465	23	7
*DLD*	42	1317	177	1317	1302	1326	84
*GGA3*	16	5	2	3	58	374	2
*IPO7*	26	2	10	1	19	443	4
*MAPK1*	33	1	3	12	2	515	1
*MIRN18A*	1314	99	695	310	574	210	1301
*MIRN148A*	567	39	204	180	20	431	164
*MIRN148B*	568	40	205	181	21	432	165
*MIRN217*	570	6	103	119	3	650	18
*MIRN375*	573	30	43	118	265	209	36
*MMP17*	366	571	493	577	575	603	380
*NDUFB5*	1322	1371	1315	1370	1371	1371	1311
*NDUFS1*	142	1313	77	1313	1298	1324	44
*PSMB9*	1336	1340	1323	1351	1326	1341	1328
*RAN*	27	4	8	2	119	444	8
*RPL6*	7	136	291	85	127	66	242
*RPL9*	28	198	235	86	128	68	259
*RPL14*	36	201	343	98	123	71	347
*RPL15*	37	228	150	94	124	73	108
*RPL31*	6	135	269	78	125	65	243
*TGFB1*	71	462	368	712	980	169	229
*UBE2C*	39	80	169	45	301	613	177
GO:0050672	1264	193	1285	299	924	47	1245
GO:0051538	1288	1350	1299	1354	1345	1350	1269
GO:0051881	1293	1321	1301	1331	1305	1330	1274

The actor nodes are listed in the alphabetical order of their gene symbols. The semiotic nodes are listed in the alphanumerical order of the Gene Ontology ID. The rank score for each centrality type ranges from 1 to 1372. A lower rank score means a higher node ranking for a particular centrality type.

## Appendix

### A.1. Representation of network G

The input for generating *G* is a set of networks {*G*_1_, …, *G*_6_} in which:

- *G*_1_(*V*_1_, *E*_1_, *ρ*) is the co-expression network where *V*_1_ is the set of *Gene* nodes, *E*_1_ is the set of pairwise gene co-expression relationships defined by the Pearson correlation (*PCC* ≥ 0.86), and *ρ* is the phenotype label which can be Coexpression_HCC or Coexpression_Liver.
- *G*_2_(*V*_2_, *E*_2_, *ɛ*) is the protein interaction network where *V*_2_ is the set of *Protein* nodes, *E*_2_ is the set of pairwise protein interactions, and *ɛ* is the interaction type which can be Human_Protein_Interaction or HBV_Human_ Interaction.
- *G*_3_(*V*_31_ ∪ *V*_32_, *E*_3_) is the microRNA regulatory network where *V*_31_ is the set of *MIRNA* nodes, *V*_32_ is the set of *Gene* nodes, and *E*_3_ is the set of predicted pairwise microRNA-to-*Gene* interactions. Given that there are two sets of inter-connected nodes, *G*_3_ is a bipartite graph.
- *G*_4_(*V*_41_ ∪ *V*_42_, *E*_4_) is the GO Process-to-Gene network where *V*_41_ is the set of *GO Process* nodes, *V*_42_ is the set of *Gene* nodes, and *E*_4_ is the set of semiotic relationships between the *GO Process* and *Gene* nodes. Given that there are two sets of inter-connected nodes, *G*_4_ is a bipartite graph.
- *G*_5_ and *G*_6_ are the *GO Function*-to-*Gene* and *GO Component*-to-*Gene* networks respectively. Their graph theoretic definition is similar to that of *G*_4_. It should be noted that *GO Component* is being classified as an actor rather than a semiotic node because it is an abstraction of a physical intracellular structure.

The output network *G* is therefore the union of *G*_1_, …, *G*_6_.

- *G* = ∪*_i_*_=1_*^k^* *G**_i_* where *k* = 6

### A.2. Representation of network G′

In addition to the set of networks listed above, the inputs for generating *G*′ are:

- The node set common to *G*_1_ and *G*_2_ is defined in the 1:1 mapping *R*_1_:*V*_11_ ↔ *V*_22_ where *V*_11_ ⊆ *V*_1_ and *V*_22_ ⊆ *V*_2_.
- The node set common to *G*_1_ and *G*_3_ is defined in the 1:1 mapping between their *Gene* nodes such that *R*_2_:*V*_11_ ↔ *V*_33_ where *V*_11_ ⊆ *V*1 and *V*_33_ ⊆ *V*_32_.
- The mapping between *G*_1_ and the individual networks *G*_4_ to *G*_6_ is similar to the definition of *R*_2_.

The output network *G*′ is therefore a result of *G*_12_ ∪ *G*_13_ ∪ *G*_14_ ∪ *G*_15_ ∪ *G*_16_ of which:

- *G*_12_(*V*_12_, *E*_12_) is derived from *G*_1_ and *G*_2_ where *V*_12_ = *V*_1_∩*V*_2_, and the edge set *E*_12_ is the subset of *E*_1_ ∪ *E*_2_, i.e.

$$E_{12}=\{e\in E_{12}|\exists e_{1}\in E_{1},e_{2}\in E_{2},e_{1}\cup e_{2}\in e\}$$

For those edges that are common to *E*_1_ and *E*_2_, i.e. *e*_1_ ↔ *e*_2_, they are being factorized to a single edge *e*_1_ but double the edge weight. Therefore *E*_12_ contains three types of edges. The first type represents both gene co-expression and pairwise protein interactions. The second type represents only gene co-expression and the last type represents only pairwise protein interactions.

- *G*_13_(*V*_131_ ∪ *V*_132_, *E*_13_) is derived from *G*_1_ and *G*_3_ where *V*_132_ is the *Gene* node set and V132 = *V*_1_∩*V*_32_. *V*_131_ is the set of *MIRNA* nodes whereas the edge set *E*_13_ is the subset of *E*_3_.
- *G*_14_(*V*_141_ ∪ *V*_142_, *E*_14_) is derived from *G*_1_ and *G*_4_ where *V*_142_ is the *Gene* node set and *V*_142_ = *V*_1_∩*V*_42_. *V*_141_ is the set of *GO Process* nodes whereas the edge set *E*_14_ is the subset of *E*_4_.
- *G*_15_ is derived from *G*_1_ and *G*_5_ whereas *G*_16_ is derived *G*_1_ and *G*_6_. The graph theoretic definition of *G*_15_ and *G*_16_ is similar to that of *G*_14_, except that *G*_15_ contains a set of *GO Function* nodes whereas *G*_16_ contains a set of *GO Component* nodes.

## References

[1] But DY Lai CL Yuen MF 2008 Natural history of hepatitis-related hepatocellular carcinoma World J. Gasteroenterol 14 1652 6 10.3748/wjg.14.1652 PMC2695904 18350595

[2] Hsu CN Lai JM Tseng HH 2007 Detection of the inferred interaction network in hepatocellular carcinoma from EHCO (Encyclopedia of Hepatocellular Carcinoma genes Online) BMC Bioinformatics 8 66 17326819 10.1186/1471-2105-8-66 PMC1828168

[3] Christensen C Thakar J Albert R 2007 Systems-level insights into cellular regulation: inferring, analyzing, and modeling intracellular networks IET Syst. Biol 1 61 77 17441550 10.1049/iet-syb:20060071

[4] Tuck DP Kluger HM Kluger Y 2006 Characterizing disease states from topological properties of transcriptional regulatory networks BMC Bioinformatics 7 236 16670008 10.1186/1471-2105-7-236 PMC1482723

[5] Chuang HY Lee E Liu YT Lee D Ideker T 2007 Network-based classification of breast cancer metastasis Mol. Syst. Biol 3 140 17940530 10.1038/msb4100180 PMC2063581

[6] Law J 1992 Notes on the theory of the actor-network: ordering, strategy, and heterogeneity Syst. Prac. Action Res 5 379 93

[7] Ieta K Ojima E Tanaka F 2007 Identification of over-expressed genes in hepatocellular carcinoma, with special reference to ubiquitin-conjugating enzyme E2C gene expression Int. J. Cancer 121 33 8 17354233 10.1002/ijc.22605

[8] Castro A Bernis C Vigneron S 2005 The anaphase-promoting complex: a key factor in the regulation of cell cycle Oncogene 24 314 25 15678131 10.1038/sj.onc.1207973

[9] Rape M Reddy SK Kirschner MW 2006 The processivity of multi-ubiquitination by APC determines the order of substrate degradation Cell 124 89 103 16413484 10.1016/j.cell.2005.10.032

[10] Ekholm-Reed S Méndez J Tedesco D 2004 Deregulation of cyclin E in human cells interferes with prereplication complex assembly J. Cell. Biol 156 789 800 10.1083/jcb.200404092 PMC2172392 15197178

[11] Bartholin L Wessner LL Chirgwin JM Guise TA 2006 The human Cyr61 gene is a transcriptional target of transforming growth factor beta in cancer cells Cancer Lett 246 230 6 16616811 10.1016/j.canlet.2006.02.019

[12] Kunz M Ibrahim SM 2003 Molecular responses to hypoxia in tumor cells Mol. Cancer 2 23 6 12740039 10.1186/1476-4598-2-23 PMC155638

[13] Perbel B 2004 CCN. proteins: multifunctional signaling regulators Lancet 363 62 4 14723997 10.1016/S0140-6736(03)15172-0

[14] Chen PP Li WJ Wang Y 2007 Expression of Cyr61, CTGF, and WISP-1 Correlates with Clinical Features of Lung Cancer PLoS ONE 2 e5 10.1371/journal.pone.0000534 PMC1888724 17579708

[15] Murakami Y Yasuda T Saigo K 2006 Comprehensive analysis of micro-RNA expression patterns in hepatocellular carcinoma and non-tumorous tissues Oncogene 25 2537 45 16331254 10.1038/sj.onc.1209283

[16] Kerbel RS 2008 Supplement to: Tumor angiogenesis N. Engl. J. Med 358 2039 49 18463380 10.1056/NEJMra0706596 PMC4542009

[17] Chung TW Lee YC Kim CH 2004 Hepatitis B viral HBx induces matrix metalloproteinase-9 gene expression through activation of ERKs and PI-3K/AKT pathways FASEB J 18 1123 5 15132991 10.1096/fj.03-1429fje

[18] Wang XW Budhu AS 2005 Loading and Unloading: orchestrating centrosome duplication and spindle assembly by Ran/Crm1 Cell Cycle 4 1510 4 16294017 10.4161/cc.4.11.2187 PMC1402358

[19] Planque N 2006 Nuclear trafficking of secreted factors and cell-surface receptors Cell. Comm. and Signaling 4 7 25 10.1186/1478-811X-4-7 PMC1626074 17049074

[20] Johnson HM Subramaniam PS Olsnes S Jans DA 2004 Trafficking and signaling pathways of nuclear localizing protein ligands and their receptors Bioessays 26 993 1004 15351969 10.1002/bies.20086

[21] Schausberger E Eferi R Parzefall W 2003 Induction of DNA synthesis in primary mouse hepatocytes is associated with nuclear pro-transforming growth factor alpha and erbb-1 and is independent of c-jun Carcinogenesis 24 835 41 12771026 10.1093/carcin/bgg027

[22] Dittmann K Mayer C Fehranbacher B 2005 Radiation-induced epidermal growth factor receptor nuclear import is linked to activation of DNA-dependent protein kinase J. Biol. Chem 280 31182 9 16000298 10.1074/jbc.M506591200

[23] Antoine M Reimers K Wirz W 2005 Fibroblast growth factor 3, a protein with a dual subcellular fate, is interacting with human ribosomal protein S2 Biochem. Biophys. Res. Commun 338 1248 55 16263090 10.1016/j.bbrc.2005.10.079

[24] Dittmann K Mayer C Rodemann HP 2005 Inhibition of radiation-induced EGFR. nuclear import by C225 (Cetuximab) suppresses DNA-PK activity Radiother. Oncol 76 157 61 16024112 10.1016/j.radonc.2005.06.022

[25] NCBI Online Mendelian Inheritance in Man (OMIM) Morbid Map http://www.ncbi.nlm.nih.gov/Omim/getmorbid.cgi

[26] Coller HA Sang L Roberts JM 2006 A new description of cellular quiescence PLoS Biol 4 e83 16509772 10.1371/journal.pbio.0040083 PMC1393757

[27] Barabási AL Oltvai Z 2004 Network biology: understanding the cell’s functional organization Nat. Rev. Genetics 5 101 13 14735121 10.1038/nrg1272

[28] Yu H Kim PM Sprecher E Trifonov V Gerstein M 2007 The importance of bottlenecks in protein networks: correlation with gene essentiality and expression dynamics PLoS Comput. Biol 3 e59 17447836 10.1371/journal.pcbi.0030059 PMC1853125

[29] Goh KI Cusick ME Valle D 2008 The human disease network Proc. Natl. Acad. Sci. U.S.A 104 8685 90 10.1073/pnas.0701361104 PMC1885563 17502601

[30] Zotenko E Mestre J O’Leary DP Przytycka TM 2008 Why do hubs in the yeast protein interaction network tend to be essential: Reexamining the connection between the network topology and essentiality PLoS Comput. Biol 4 e1000140 18670624 10.1371/journal.pcbi.1000140 PMC2467474

[31] Beerenwinkel N Antal T Dingli D 2007 Genetic progression and the waiting time to cancer PLoS. Comput. Biol 3 e225 17997597 10.1371/journal.pcbi.0030225 PMC2065895

[32] Cui Q Ma Y Jaramillo M 2007 A map of human cancer signaling Mol. Syst. Biol 3 152 18091723 10.1038/msb4100200 PMC2174632

[33] Vogelstein B Kinzler KW 2004 Cancer genes and the pathways they control Nat. Med 10 789 99 15286780 10.1038/nm1087

[34] Cui Q Yu Z Purisma EO Wang E 2006 Principles of microRNA regulation of a human cellular signaling network Mol. Syst. Biol 2 46 16969338 10.1038/msb4100089 PMC1681519

[35] Legewie S Blüthgen N Schäfer R Herzel H 2005 Ultra-sensitization: switch-like regulation of cellular signaling by transcriptional induction PLoS. Comput. Biol 1 e54 16261195 10.1371/journal.pcbi.0010054 PMC1274294

[36] Gamberoni G Storari S Volinia S 2006 Finding biological process modifi cations in cancer tissues by mining gene expression correlations BMC Bioinformatics 7 6 16401337 10.1186/1471-2105-7-6 PMC1360676

[37] Chen X Cheung ST So S 2002 Gene expression patterns in human liver cancers Mol. Biol. Cell 13 1929 39 12058060 10.1091/mbc.02-02-0023. PMC117615

[38] Luo F Yang Y Zhong J 2007 Constructing gene co-expression networks and predicting functions of unknown genes by random matrix theory BMC Bioinformatics 8 299 17697349 10.1186/1471-2105-8-299 PMC2212665

[39] Bandrés E Cubedo E Agirre X 2006 Identification by real-time PCR of 13 mature microRNAs differentially expressed in colorectal cancer and non-tumoral tissues Mol. Cancer 5 29 16854228 10.1186/1476-4598-5-29 PMC1550420

[40] Szafranska AE Davison TS John J 2007 MicroRNA expression alterations are linked to tumorigenesis and non-neoplastic processes in pancreatic ductal carcinoma Oncogene 26 1 11 17237814 10.1038/sj.onc.1210228

[41] Xi Y Edwards J Ju J 2007 Investigation of miRNA biology by bioinformatics tools and impact of miRNAs in colorectal cancer—regulatory relationship of c-Myc and p53 with miRNAs Cancer Informatics 3 245 53 18079974 PMC2133370

[42] Gene Ontology Consortiu 2006 The Gene Ontology (GO) project in 2006 Nuclei Acids Res. (database issue) 34 D322 326 10.1093/nar/gkj021 PMC1347384 16381878

[43] Stark C Breitkreutz BJ Reguly T 2006 BioGRID: a general repository for interaction datasets Nuclei Acids Res 34 D535 539 10.1093/nar/gkj109 PMC1347471 16381927

[44] Borgatti S 2004 Lecture notes MB.101 Emergent groups

[45] Eckmann JP Moses E 2002 Curvature of co-links uncovers hidden thematic layers in the world wide web Proc. Natl. Acad. Sci. U.S.A 99 5825 9 11972019 10.1073/pnas.032093399 PMC122861

[46] Junker BH Koschützki D Schreiber F 2006 Exploration of biological network centralities with CentiBiN BMC Bioinformatics 7 219 16630347 10.1186/1471-2105-7-219 PMC1524990

[47] Estrada E 2006 Virtual identification of essential proteins within the protein interaction network of yeast Proteomics 6 35 40 16281187 10.1002/pmic.200500209

[48] Venables WN Ripley BD 2002 Modern Applied Statistics with S 4 Springer Press

